# Supplementary material for: Scalable intracellular delivery via microfluidic vortex shedding enhances the function of chimeric antigen receptor T-cells
Source: Sci Rep. 2025 Feb 17;15:5749. doi: 10.1038/s41598-025-89070-5 (PMC11832915; doi:10.1038/s41598-025-89070-5)
Supplement: Supplementary file 1 — Supplementary Material 1 [file 41598_2025_89070_MOESM1_ESM.pdf]

Contents

Supplemental figures

1. Computational fluid dynamics simulations and related biological data for different flow cell widths - Supplemental Fig. 1
2. Representative flow cytometry gating for assessing TCR surface expression following Cas9 TRAC-RNP delivery to and CAR AAV transduction of CD3+ T cells, TCR KO% and KIKO ratios - Supplemental Fig. 2
3. Representative flow cytometry gating for assessing CD62L/CD45RA phenotypes.- Supplemental Fig. 3
4. Additional single-cell TIMING data for the five donors - Supplemental Fig. 4
5. Bioluminescence imaging trace for each mouse - Supplemental Fig. 5
6. Representative flow cytometry gating for TCR/EGFR surface expression before and after magnetic TCR depletion ahead of *in vitro* NALM6 co-culture or infusion into mice. - Supplemental Fig. 6

Supplemental tables

1. Antibodies used for flow cytometry analysis - Supplemental Table 1

Supplemental videos

1. Montage and video of individual time points (hh:mm) during a TIMING assay - Supplemental Video 1

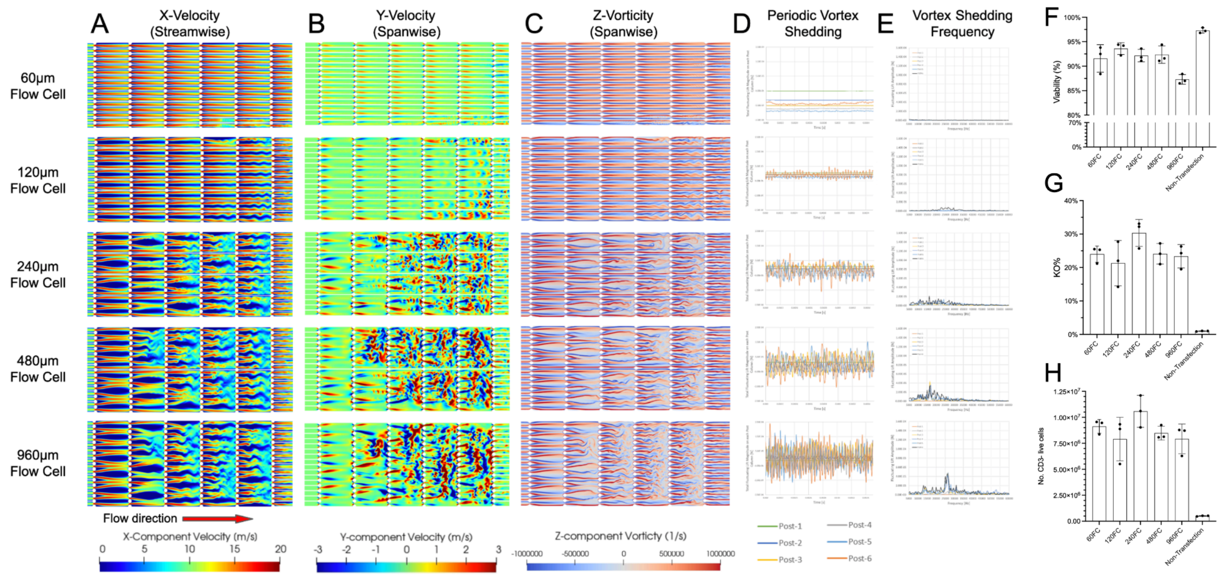

Supplemental Fig. 1 | ** Computational fluid dynamics simulations and related biological data for different flow cell widths showing **A) x-velocity, **B)** y-velocity, **C)** z-vorticity, **D)** time-domain force acting on individual posts, **E)** frequency-domain force acting on individual posts, **F)** cell viability at 24 hr, **G)** TRAC-1 KO efficiency on day 7, and **H)** TRAC-1 KO yield on day 7. All data points for f-h involve n = 3 biological data points and the error bars represent 1 standard deviation. Optimal 240 µm flow cell width equivalent to 1x flow cell and data related to Fig 2a,b.

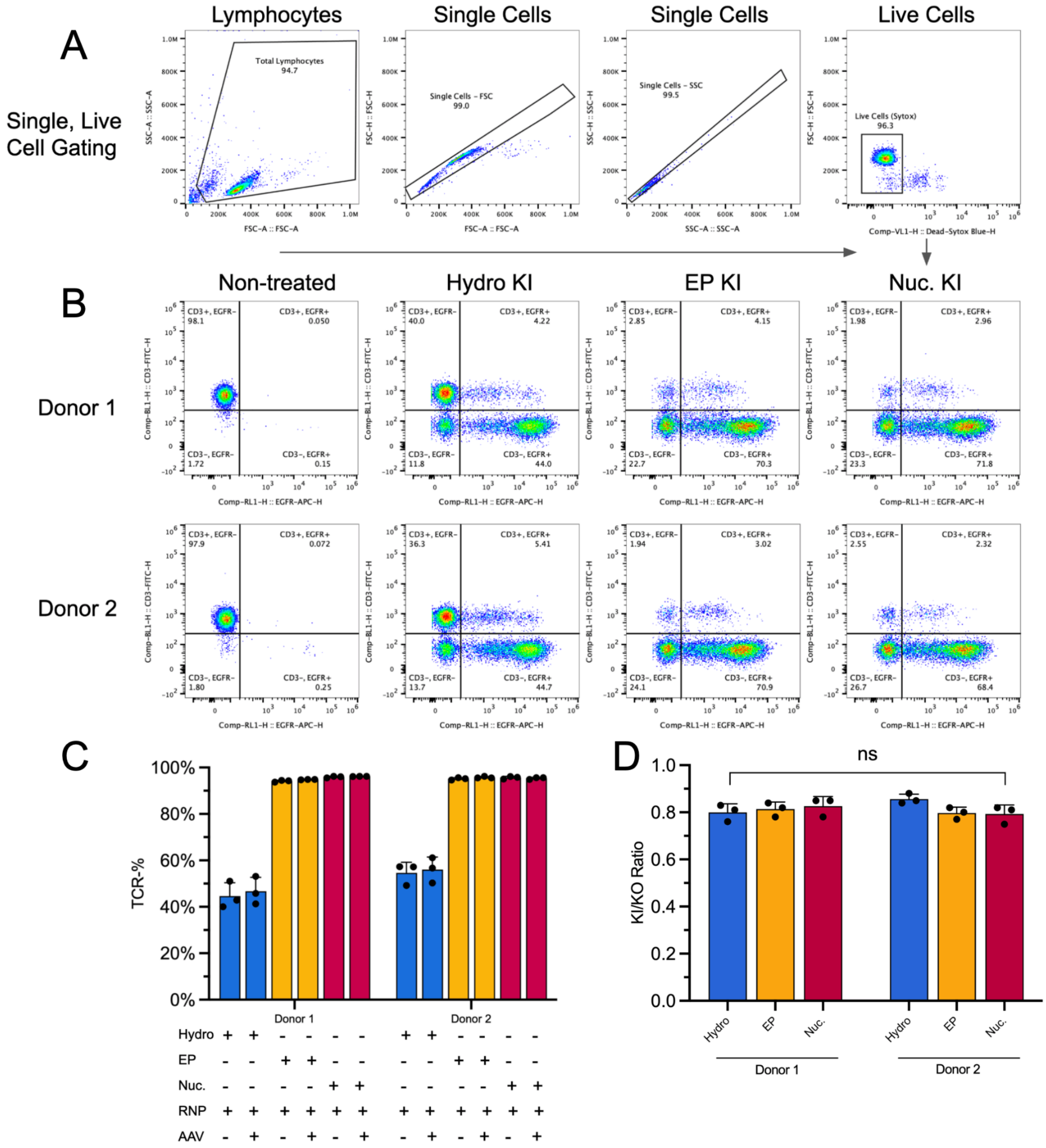

Supplemental Fig. 2 | Representative flow cytometry gating for assessing TCR surface expression following Cas9 TRAC-RNP delivery to and CAR AAV transduction of CD3+ T cells for **A)** Singe, live cell gating strategy **B)** Representative TCR KO/EGFR KI flow plots for three different transfection methods. Data are related to Fig 1g,h **C)** TCR knock-out efficiencies for the three different transfection methods +/- the addition of the AAV HDRT. **D)** The ratio of knock-in efficiency to knockout efficiency. Statistical significance by Brown-Forsythe ANOVA test, n=3, p= 0.32.

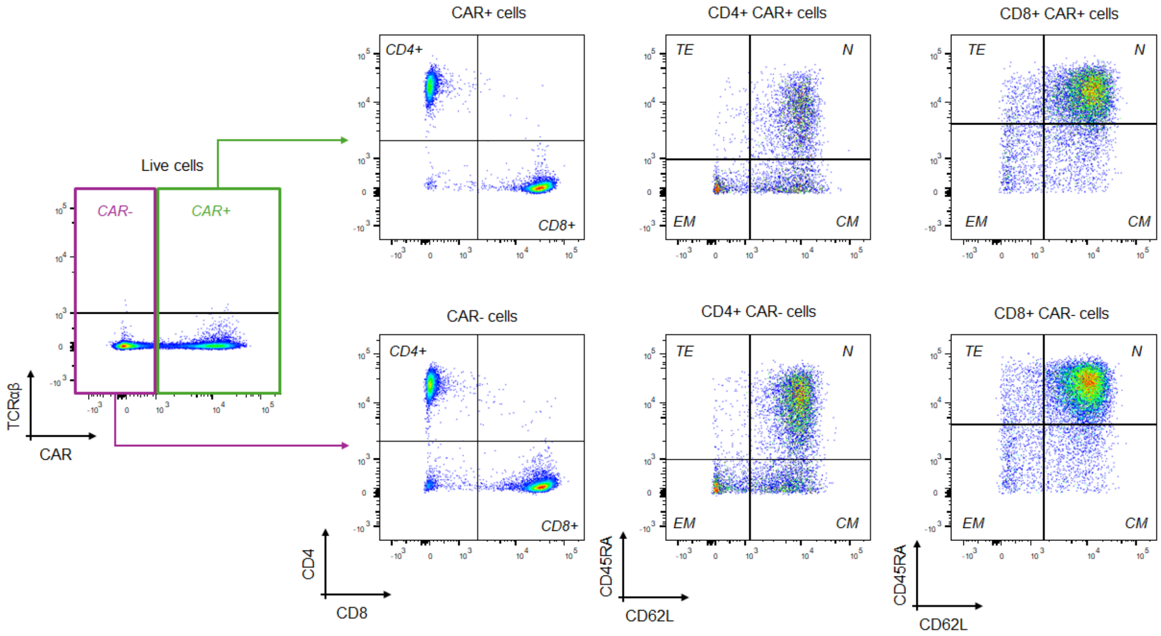

Supplemental Fig. 3 | Representative flow cytometry gating for assessing CD62L/CD45RA phenotypes in CD4+ and CD8+ T cells. Phenotypes were assessed independent of editing outcome. Data related to Fig 3a.

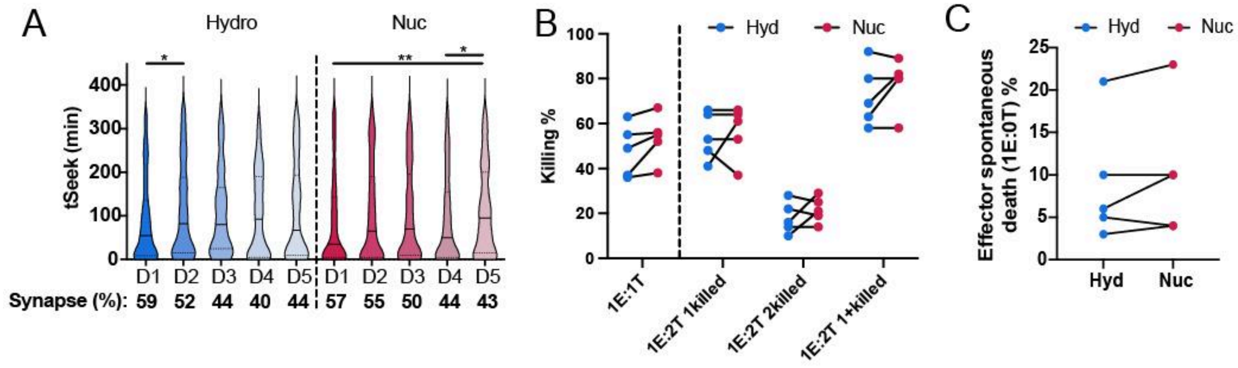

Supplemental Fig. 4 | Additional single-cell TIMING data for the five donors shown in Fig. 4. **A)** tSeek (time for CAR-T cells to form a synapse with a target cell). The percentage of CAR-Ts that formed a synapse with one target (1E:1T nanowells) is shown below the x-axis. **B)** Percent of CAR-Ts that formed a synapse and killed one or more targets after synapse formation. **C)** 0T nanowells (1 CAR-T: 0 target cells = no target cells present) that were dead after the six-hour assay. Statistical tests were performed and denoted as described in Fig. 4.

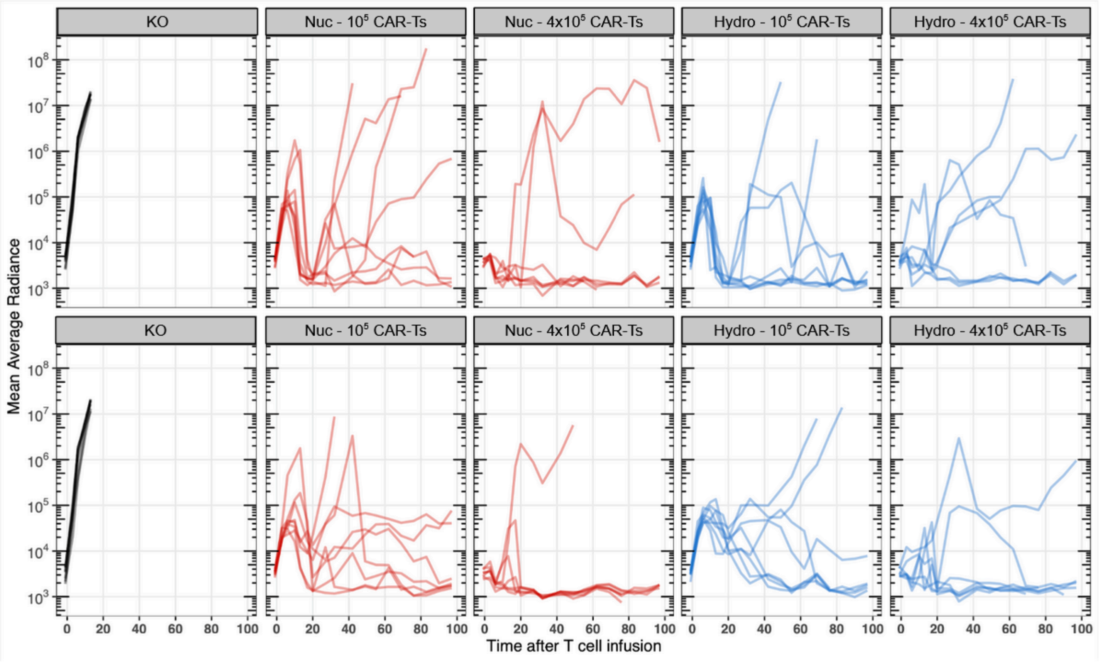

Supplemental Fig. 5 | Bioluminescence imaging trace for each mouse associated with data in Fig. 5c.

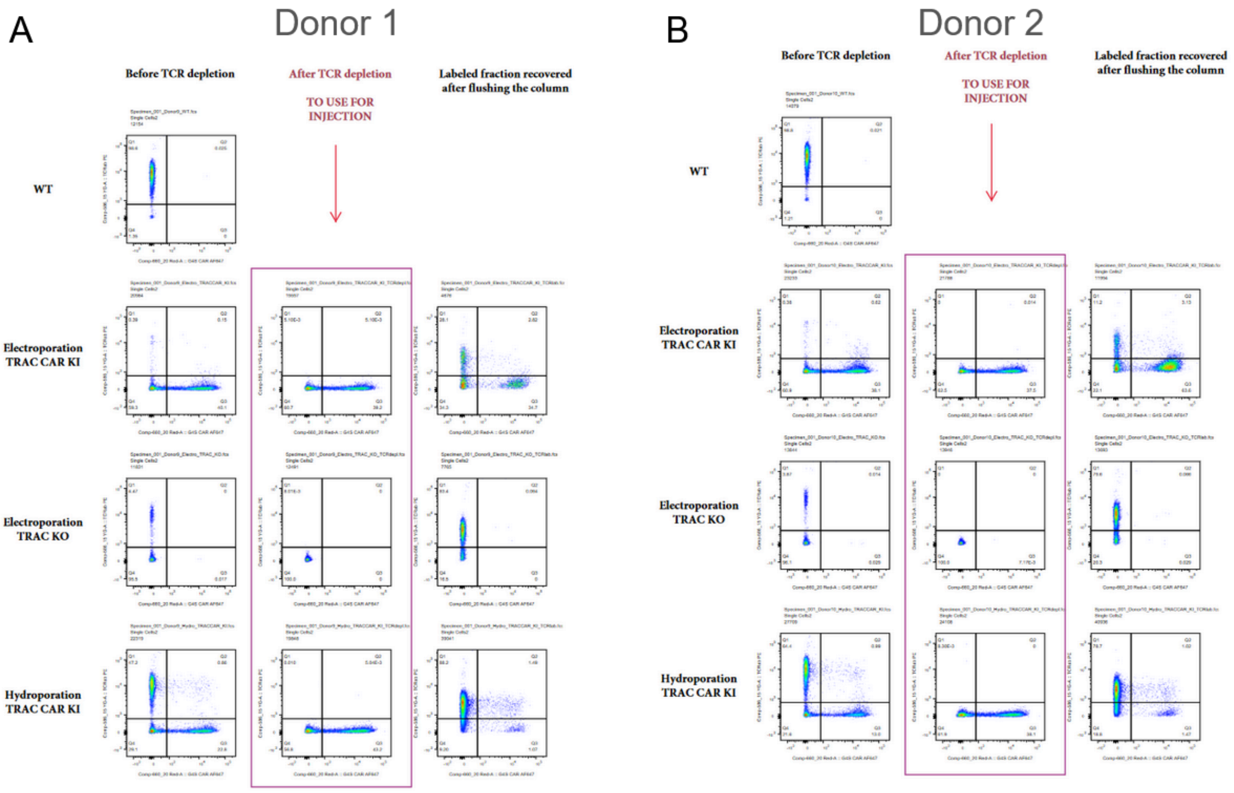

Supplemental Fig. 6 | Representative flow cytometry gating for assessing TCR/EGFR surface expression following Cas9 TRAC-RNP delivery to and CAR AAV transduction of CD3+ T cells for **A)** Donor 1 and **B)** Donor 2. Data are related to Fig 5.

Supplemental Table. 1 | Antibodies/stains used for flow cytometry analysis.

Target	Fluorophore	Dilution	Manufacturer	Clone	Catalog #
CD3	AF700	1:100	Thermo Fisher Scientific	UCHT1	56-0038-42
CD3	FITC	1:100	BioLegend	UCHT1	300405
CD4	PE-Cy7	1:500	BioLegend	OKTA	317413
CD8	PE-Texas Red	1:500	Thermo Fisher Scientific	3B5	MHCD0817
PD-1	APC	1:200	Thermo Fisher Scientific	J105	17-2799-41
Lag3	PE	1:100	Thermo Fisher Scientific	3DS223H	12-2239-41
CD27	FITC	1:100	Thermo Fisher Scientific	O323	11-0279-42
CD197 (CCR7)	APC-eF780	1:200	Thermo Fisher Scientific	3D12	47-1979-42
CD45RA	PerCP-Cy5.5	1:200	Thermo Fisher Scientific	H100	45-0458-41
TCRα/β	FITC	1:200	Miltenyi Biotec	REA652	130-113-538
EGRP	APC	1:200	BioLegend	AY13	352906
n/a	GhostDye Red 780	1:1000	Tonbo Biosciences	n/a	13-0865-T500
n/a	Sytox Blue	1:1000	Thermo Fisher Scientific	n/a	S34857
n/a	Propidium Iodide (PI)	1:1000	Thermo Fisher Scientific	n/a	P4170

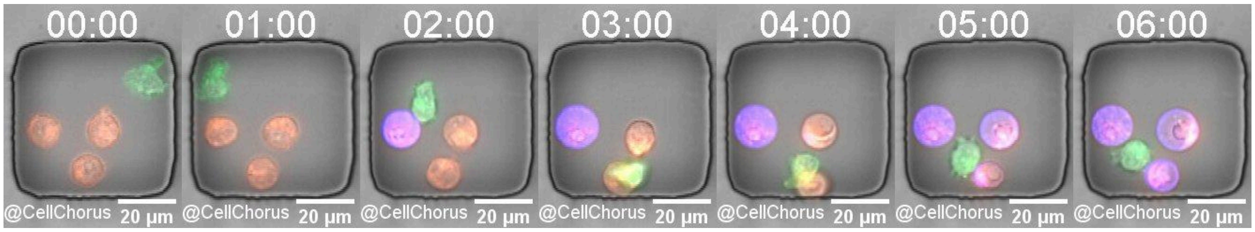

Supplemental Video. 1 | Montage and video of individual time points (hh:mm) during a TIMING assay of Hyd-CAR-T (green) interacting with three NALM6 target cells (red) in a nanowell. Apoptotic cells are identified with AnnexinV-Alexa674 (purple) during the assay. This Hyd-CAR-T is a serial killer and killed all three target cells in the nanowell within 6 hrs.
